# Bioactive Compounds in Food as a Current Therapeutic Approach to Maintain a Healthy Intestinal Epithelium

**DOI:** 10.3390/microorganisms9081634

**Published:** 2021-07-30

**Authors:** Eva Salinas, Diana Reyes-Pavón, Naima G. Cortes-Perez, Edgar Torres-Maravilla, Oscar K. Bitzer-Quintero, Philippe Langella, Luis G. Bermúdez-Humarán

**Affiliations:** 1 Department of Microbiology, Basic Science Center, Autonomous University of Aguascalientes, Aguascalientes 20100, Mexico; emsalin@correo.uaa.mx (E.S.); nutrois@gmail.com (D.R.-P.); 2 Université Paris-Saclay, INRAE, AgroParisTech, UMR 0496, 78350 Jouy-en-Josas, France; naima.cortes-perez@inrae.fr; 3 Université Paris-Saclay, AgroParisTech, Micalis Institute, INRAE, 78350 Jouy-en-Josas, France; edgar.torres-maravilla@inra.fr (E.T.-M.); philippe.langella@inrae.fr (P.L.); 4 Neurosciences Division, Centro de Investigación Biomédica de Occidente, Instituto Mexicano del Seguro Social, Guadalajara 44340, Mexico; neuronim26@yahoo.com

**Keywords:** functional food, probiotics, prebiotics, synbiotics, IBD, IBS, FA

## Abstract

The intestinal epithelium serves as an effective barrier against the external environment, hampering the passage of potentially harmful substances (such as pathogenic microbes) that could trigger an exacerbated host immune response. The integrity of this barrier is thus essential for the maintenance of proper intestinal homeostasis and efficient protective reactions against chemical and microbial challenges. The principal consequence of intestinal barrier defects is an increase in intestinal permeability, which leads to an increased influx of luminal stressors, such as pathogens, toxins, and allergens, which in turn trigger inflammation and immune response. The fine and fragile balance of intestinal homeostasis can be altered by multiple factors that regulate barrier function, many of which are poorly understood. This review will address the role of gut microbiota as well as food supplements (such as probiotics, prebiotics, and synbiotics) in modulating gut health and regulating intestinal barrier function. In particular, we will focus on three human pathologies: inflammatory bowel disease, irritable bowel syndrome, and food allergy.

## 1. Introduction

The gastrointestinal tract (GIT) is the largest mucosal surface of the human body that is in continuous contact with the external environment. It is covered by a single layer of cells that provides a biochemical and physical barrier between the host and the external environment, thus protecting the host from potentially irritating and antigenic substances present in the luminal compartment [1]. The epithelial layer that constitutes this barrier is regulated by a network of proteins that orchestrates complex biological functions, such as absorption, secretion, and transport of various nutrients and water [2]. Selective permeability is provided by the paracellular pathway and mediated by the junctional protein complex, which connects epithelial cells to each other. Intestinal barriers represent a dynamic and responsive interface and play a crucial role in maintaining intestinal homeostasis and thus host health [2]. The GIT is also colonized by a large number of microorganisms living in symbiosis with their host, collectively known as the microbiota (formerly microflora); these microorganisms are now widely recognized as a key regulator of intestinal homeostasis and overall gut health, with numerous beneficial effects [3].

A healthy gut barrier prevents tissue injury and pathogen spread and thus hinders the development of diseases. When the function of the intestinal barrier is compromised, potentially harmful microbes are able to pass through (i.e., leaky gut) [4]. In this context, there is great interest in identifying the factors and conditions that influence intestinal barrier function, as they may have a profound impact on several intestinal disorders, including inflammatory bowel diseases (IBD) [5], irritable bowel syndrome (IBS) [6], and colorectal cancer [7], as well as on other non-gastrointestinal pathologies, such as obesity [8], diabetes [9], neurological disorders [10], and food allergies (FA) [11,12].

The gut is a multifaceted milieu exposed to numerous alimentary components and commensal bacteria; the interactions within this environment are diverse and often synergistic. For example, the epithelium shapes microbial communities by producing mucin and antimicrobial factors that limit bacterial colonization and adherence. The composition of the diet (such as intake of prebiotics) can also have an impact on the gut epithelial barrier and its associated bacterial communities [13]. On the other hand, gut microbiota can play a key role in intestinal health by maintaining the integrity of the intestinal barrier, modulating the immune system, and assisting host metabolism. Microbiota or bacteria originating from food (such as probiotics) secrete active compounds that are by-products of their metabolism, such as essential vitamins, antioxidants, and short-chain fatty acids (SCFAs), which affect host homeostasis [1]. A slight alteration in the composition of gut microbiota (i.e., dysbiosis) can lead to diverse pathophysiological conditions [14]. In addition, it is well established that dietary components play beneficial roles for the host that go beyond basic nutrition, a feature that has led to the development of functional food concepts [15,16]. For instance, several food supplements have been designed around the consumption of either live bacteria (i.e., probiotics) or substrates specifically used by host microorganisms (such as prebiotics), which together are the best-characterized bioactive compounds in the diet and have been shown to have a beneficial impact on gut health and overall host well-being [15,17]. In this review, we describe the roles of the intestinal epithelium and gut microbiota in mucosal immunity and the influence of certain functional food components in modulating intestinal health, with special emphasis on probiotics, prebiotics, and synbiotics in IBD, IBS, and FA pathological conditions.

## 2. Composition and Function of the Intestinal Epithelial Barrier

**Figure 1:**
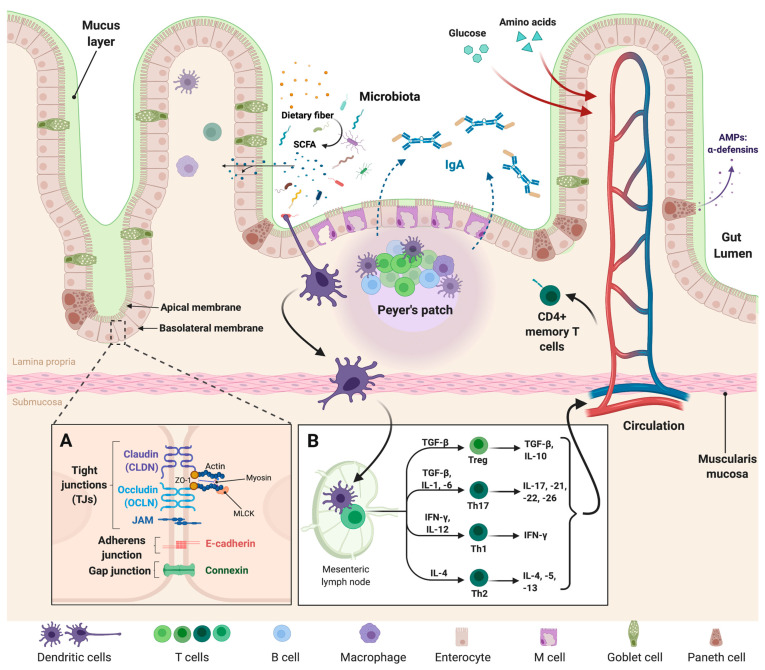
Intestinal epithelial barrier organization. The intestinal epithelium is composed of enterocytes, enteroendocrine cells, M cells, Goblet cells, and Paneth cells. (**A**) Structure and composition of the junctional protein complex: the junctional protein complex consists mainly of tight, adherens and gap junctions; they seal the intracellular space and regulate paracellular permeability. (**B**) The GIT is also closely related to the local immune system: the immune response is induced through the expression of TLRs on macrophages, dendritic cells, and T- and B-lymphocytes. This response increases the production of secretory immunoglobulin A (IgA) associated with the action of M-cells to B-lymphocytes. This IgA is able to distinguish between commensal and pathogenic bacteria and thus maintain the balance and regulation of intestinal homeostasis. Diets containing fermentable fibers and resistant starches result in an increase in intestinal fermentation (by bacteria present in gut microbiota, mainly), and therefore to an increase in the production of short-chain fatty acids (SCFAs), such as acetate, butyrate, and propionate. On the other hand, glucose and amino acid molecules issued from diet diffuse from the small intestine lumen into blood capillaries. (This figure was created with Biorender.com, access date: 24 May 2021).

The intestinal epithelium is constantly in a state of self-renewal, with proliferative stem cells that engender several specific cell lineages, including enterocytes, goblet cells, entero-endocrine cells, and Paneth cells [18,19,20]. Although the distribution and proportion of these cells throughout the GIT vary, collectively, they protect the host against dangerous luminal substances. Indeed, the main function of the intestinal barrier is to regulate nutrient passage and water absorption while protecting the host from the entry of substances harmful to lamina propria (LP) [19]. It comprises both a chemical barrier, formed by the mucosa and immune system components and a physical first line of defense composed of cells and mucus layers. The epithelial cell lining facilitates gastrointestinal transport and acts as a protective layer against bacterial invasion; this is covered by the mucus layer, which contains secretory immunoglobulin (Ig) A and antimicrobial peptides (AMPs), such as α-defensins (Figure 1). The mucus layer of the colon is composed of two layers, an outer and an inner layer, both formed of highly glycosylated gel-forming proteins called mucins. These are produced and maintained by goblet cells that renew the inner mucus layer approximately every hour [18]. These dynamic processes are subjected to extensive and continuous interaction with the intestinal microbiota, the alteration of which may have implications for the maintenance of key barrier functions. Physical interactions between cells, mediated by tight junction (TJ) structures formed by proteins, such as claudin (CLDN), and occludin (OCLN), also play an important role. These transmembrane proteins connect to the neighboring plasma membrane to form a mechanical link between epithelial cells and thus, establish a barrier to the paracellular diffusion of fluids and solutes [20].

## 3. Gut Microbiota, the Microbiome, and the Intestinal Epithelium

The GIT harbors one of the most complex ecosystems described to date: the gut microbiota. The microbiota is defined as the entire population of microbes present in the human body; it is dominated by bacteria but also includes archaea, eukaryotes, and viruses [21,22]. The human GIT can harbor about 10^14^ microorganisms, a number almost three-fold greater than the total number of human cells in the entire body [23]. The human gut microbiota is composed of approximately 600 different bacterial species, 98% of which originate from two dominant phyla, Bacteroidetes and Firmicutes (25). In addition, it is well established that the number of genes in the gut microbiota (i.e., the microbiome: over 22 million genes) is approximately 10-fold larger than that of the human genome itself and appears to represent a co-evolutionary relationship [24,25]. Due to the abundant interactions among different bacterial species, human host cells, and the external environment, the microbiota can be conceptualized as a dynamic ecological community [26]. In this sense, the dynamic balance of the gut microbiota in the human body is critical for health, and this can be altered by environmental factors, external stimuli, and physiological changes, such as antibiotic use, disease, stress, aging, poor dietary habits, and lifestyle [26]. These alterations often result in an imbalance in the microbial community (i.e., dysbiosis), with direct associations to multiple pathological conditions.

Several studies have demonstrated associations between modifications in the composition of gut microbiota and different intestinal barrier disorders; indeed, dysbiosis has been frequently observed in many chronic diseases of the intestine as IBD [27] and IBS [28]. Links have also been found with many non-gastrointestinal pathologies, such as obesity, diabetes, FA, autism, and Parkinson’s disease [29,30].

## 4. The Intestinal Epithelium: At the Interface of Gut Microbiota and Mucosal Immunity

The GIT contains the largest number of lymphoid cells of the entire immune system and thus represents the largest immune organ in the human body [31]. The components of innate and adaptive immunity are mainly localized in the LP and are able to prevent excessive inflammatory responses. These immune responses are driven by macrophages and dendritic cells (DCs). Indeed, commensal or pathogenic bacteria that access the lymphoid tissue are rapidly phagocytized by intestinal macrophages present in the LP. The innate response is induced by the recognition of molecular patterns by receptors expressed on intestinal epithelial cells, macrophages, DCs, and B and T lymphocytes [31]. The most well-known of these are the Toll-like receptors (TLRs), whose signaling pathway is involved in maintaining the integrity of the intestinal epithelium. The adaptive immune response is largely regulated by DCs located in the lymphoid tissue, which is able to reach the epithelium to sample the contents of the intestinal lumen and present antigens to T-cells in the follicles and lymphoid tissue [32]. This process occurs throughout the GIT, but the specialized sites of this response are known as Peyer’s patches (Figure 1). Antigen transport across the epithelium also occurs through the action of M cells, whose “pocket” at the basal pole contains B lymphocytes that are associated with IgA secretion [33]. These non-inflammatory antibodies are the ones most commonly found among mucosal secretions [34]. They are recognized by polymeric immunoglobulin receptors present on the surface of intestinal epithelial cells, which then facilitate the passage of IgA through the epithelial cells into the intestinal lumen (Figure 1). In the mucus layer, IgA distinguishes between commensal and pathogenic bacteria, allowing the maintenance of the former while neutralizing the latter via different mechanisms that reduce their adhesion properties and mobility. The regulation of intestinal homeostasis is thus achieved through continuous mechanisms of dialogue between the mucosa-associated immune system and the microbiota [35].

As mentioned above, alterations in the integrity of the epithelial barrier are associated with a wide range of gastrointestinal and non-gastrointestinal disorders [5,6,7,8,9,10,11,12]. The passage of pro-inflammatory molecules and harmful pathogenic microorganisms through the impaired intestinal barrier induces the activation of the immune system, which can lead to the appearance of processes associated with chronic inflammation [36]. It is now clear that most chronic syndromes or diseases are associated with a leaky gut and low-grade inflammation. Pro-inflammatory cytokines, such as TNF-α or IFN-γ, have also been shown to play a role in the regulation of junctional proteins, inducing a vicious cycle in the leaky gut process [36,37]. However, intestinal permeability can also be affected by other factors, such as psychological stress, heredity, nutrition, age, or lifestyle. It is, therefore, still difficult to know whether intestinal hyperpermeability is the cause or the consequence of either intestinal diseases or other non-gastrointestinal pathologies [38].

As a step toward the development of better therapeutic approaches for the treatment of these pathologies, it is first necessary to improve our understanding of epithelial homeostasis and its pathogenesis, as well as the functions of the intestinal barrier.

## 5. Modulation of Intestinal Permeability by Food Supplements: Probiotics, Prebiotics and Synbiotics

Thus, as discussed above, the integrity of the intestinal barrier is essential for mucosal homeostasis, and recent evidence suggests the beneficial effects of supplementing food with host health-promoting bacteria (e.g., probiotics), non-viable components that confer a health benefit when specifically used by microorganisms (e.g., prebiotics), and combinations of the two (synbiotics) [38].

Probiotics have been defined as “live microorganisms, which when ingested in sufficient quantity, provide a health benefit to the host” [13]. Probiotics have demonstrated potential beyond simple modulation of the gut microbiota; their beneficial effects have been demonstrated both in vitro and in preclinical trials, particularly in the case of some *Lactobacillus* and *Bifidobacterium* strains, which have been by far the best documented probiotic genera. The health benefits associated with the consumption of probiotics have also been studied with the aim of finely tuning the strain(s) used for the treatment of different pathologies, such as obesity, cancer, and depression. In addition, these microorganisms are also able to display immunomodulatory activities and to secrete functional molecules, such as enzymes, SCFAs (e.g., acetate, propionate, and butyrate), and vitamins, among many others, which provide more generalized health benefits to the host [39].

Recent research has gone further by investigating the health impact of prebiotics, which are substrates that are selectively utilized by host microorganisms and which may confer health benefits [17]. To date, the most widely documented prebiotics with health benefits are: inulin, fructo-oligosaccharides (FOS), oligofructose, galacto-oligosaccharides (GOS), and lactulose [40]. Other fermentable carbohydrates that have demonstrated prebiotic potential include resistant starch, β-glucans, arabinoxylan oligosaccharides, xylo-oligosaccharides, soybean oligosaccharides, isomalto-oligosaccharides, and pectin [41]. Sources of prebiotics are mainly found naturally occurring in foods, such as breads, cereals, onions, garlic, and artichokes, but they can also be ingested in the form of dietary supplements [42].

Finally, there has been great interest in recent years in the use of probiotics in combination with prebiotics, collectively referred to as synbiotics, in which both components have demonstrated health benefits in the host [43]. Currently, synbiotics are defined as a mixture comprising live microorganisms and substrates selectively utilized by host microorganisms that confers a health benefit to the host [44]. The term “host microorganisms” in this context includes both autochthonous (commensal) and allochthonous (externally applied, such as probiotics) microorganisms. Along these lines, synbiotics can be classified as synergistic or complementary; in the former case, the substrate is designed to be used selectively by the co-administered microorganism, and in the latter, the prebiotic component is designed to target autochthonous microorganisms [44].

Since dysbiosis is common in patients with IBD, IBS, and FA, probiotics and prebiotics have become attractive and promising agents of host-microbiome modulation therapies for various diseases, including IBD, IBS, and FA [43,45,46], demonstrating beneficial effects in ameliorating disease complications; besides, the administration of synbiotics may improve the efficacy of treatment more than the use of one of the components alone.

## 6. Intestinal Epithelial Barrier Disorders

### 6.1. Inflammatory Bowel Diseases

IBD is a group of increasingly prevalent disorders of the GIT that includes ulcerative colitis (UC) and Crohn’s disease (CD) [45]. These pathologies are characterized by reduced bacterial diversity in the gut and marked changes in the abundance of certain bacterial taxa, such as a decreased abundance of Bacteroides, Firmicutes, *Lactobacillus*, and Ruminococcaceae, and increased abundance of Gamma-proteobacteria and Enterobacteriaceae, along with altered microbiome-associated metabolite profiles [46,47]. Disruption of the tightly regulated intestinal barrier can result in the introduction of bacteria to the mucosal layer, leading to enhanced host immune responses and tissue injury [48,49]. In this way, alterations in the integrity of the intestinal barrier, including the mucus layer, epithelial cell junctions, and AMP secretion, are thought to contribute to IBD pathogenesis [50].

IBD disorders are confined to the colon for UC and to the colon and small intestine for CD. Both diseases have periods of remission and relapse with clinical symptoms of diarrhea, abdominal pain, intestinal bleeding, and weight loss [45]. Mechanistically, diarrhea is the result of an imbalance between electrolyte absorption and/or secretion in the intestines that induces water retention in the intestinal lumen [51]. In patients with these conditions, intestinal barrier dysfunction has been observed, such as a decrease in the thickness of the mucus layer and deficient production of AMPs [52]. In addition, IBD has been associated with a decline in the population of Firmicutes and an increase in Gram-negative bacteria in fecal samples of patients [53]. For instance, an increase in potentially pro-inflammatory bacteria, such as *Escherichia coli*, and a decrease in anti-inflammatory bacteria, such as *Faecalibacterium prausnitzii*, have been observed in the intestinal microbiota of patients with CD [54]. *F. prausnitzii*, a commensal intestinal bacterium known for its anti-inflammatory activity, is able to alleviate IBD symptoms in experimental colitis murine [55], although the mechanism employed by this bacterium remains to be elucidated. Nowadays, there is evidence that a single microbial anti-inflammatory molecule (MAM) identified in *F. prausnitzii* supernatants (SN), inhibits the NF-κB pathway and prevents colitis in a murine model [56]. In addition, butyrate produced by this strain shows anti-inflammatory properties through the upregulation of *Dact3* (see below), a gene linked to the Wnt/JNK pathway [57]. The loss of microbial butyrate, an important energy source for colonocytes, may adversely affect the progression of IBD. According to Sitkin et al. [58], butyrate supplementation to mesalazine treatment in patients with active UC results in an increase in butyryl-CoA:acetate CoA-transferase gene content in the fecal microbiota, a decrease in the higher *Bacteroides fragilis*/*F. prausnitzii* ratio, and an improvement of disease symptoms.

**Figure 2:**
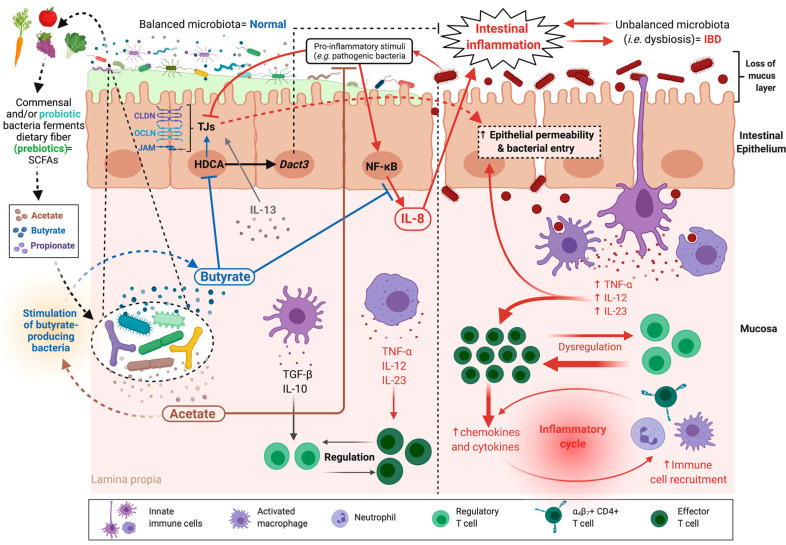
Increased intestinal permeability in inflammatory bowel diseases (IBD). In IBD-related disorders, the intestinal permeability is increased, the thickness of the mucus layer is decreased, and normal microbiota is unbalanced, resulting in bacteria entry to mucosa and activation of the mucosal immune system. Significant dysregulation of cytokine and chemokine levels is observed, which induces an inflammatory cycle. Pro-inflammatory stimuli induced by pathogenic bacteria, together with IL-13 produced by the inflammatory cells of the mucosa, dysregulate tight junction (TJ) protein expression, which enhances intestinal hyperpermeability, and activate enterocytes to secrete IL-8 and other inflammatory mediators. Probiotic bacteria, their by-products, such as the short-chain fatty acids (SCFAs) butyrate and acetate, and prebiotics modulate dysbiosis and restore a balanced microbiota. They also skew the immune microenvironment to an anti-inflammatory profile. The main target molecules of these therapies in the treatment of IBD are indicated. (This figure was created with Biorender.com, access date: 24 May 2021).

In IBD, dysbiosis induces an increase in intestinal permeability, which results in the activation of the mucosal immune system through the translocation of antigens, which then perpetuates the inflammation. However, whether gut alteration is the primary cause of disease or the consequence of predominant inflammation, has not yet been established [59]. One important factor that has emerged from IBD studies (most notably in patients with CD) is an increased abundance of claudins that are responsible for paracellular selectivity, specifically claudin-2 [60]. Claudin-2 expression is attributed to the presence of cytokines, such as IL-13 [61], and is a major contributor to increased permeability (Figure 2). However, there is emerging evidence that claudin-2 may also play a protective role as a signal modulator that accelerates the cell proliferation process and blocks pro-inflammatory cytokines [62]. Activation of the myosin light chain kinase (MLCK) via TNF-α production has also been implicated in increased permeability [63]. MLCK plays a role not only in the direct regulation of the cytoskeleton but also in the production of IL-13, which then increases claudin-2 expression [64]. Different expression patterns have instead been observed for other junctional proteins; decreased expression was noted for claudins 4 and 7 and the JAM protein, while claudins 5 and 8 seem to be redistributed [60,65]. In this context, several commensal and probiotic bacteria have been shown to protect intestinal barrier function and enhance intestinal barrier repair. Indeed, strains of *Lactobacillus acidophilus* and *Bacteroides thetaiotaomicron* prevent the increase in permeability induced by pro-inflammatory cytokines [66], while strains of *F. prausnitzii* and *Lactobacillus rhamnosus* normalize the increase in colonic permeability in a murine model of DNBS-induced colitis [67]. This improvement was due to a partial restoration of intestinal barrier function and increased levels of the TJ protein OCLN [67]. Interestingly, *F. prausnitzii* is one of the most abundant butyrate-producing commensal bacteria (see above) [54]. This SCFA plays an important role in intestinal physiology and has pleiotropic effects on the life cycle of intestinal cells and numerous positive health effects [54]. Furthermore, butyrate has been proposed to have anti-inflammatory activities [68]. Indeed, in a recent study, the authors showed that treatment of human intestinal epithelial cells (HT-29) stimulated with TNF-α, with either *F. prausnitzii* culture SN (which contains high levels of butyrate) or butyrate, decreased the secretion of the pro-inflammatory cytokine IL-8 [57]. Moreover, butyrate binds to histone deacetylases (HDACs), causing their inhibition, a phenomenon that results in the induction of *Dact3* expression, a gene that seems to play an important role in blocking IL-8 [57] (Figure 2). Furthermore, butyrate-induced hyperacetylation allows the synthesis of epithelial cell TJs proteins, such as JAM, CLDNs, and OCLN [69] (Figure 2). Another interesting study clearly shows that acetate-producing bifidobacteria acts *in vivo* to promote the defense functions of host epithelial cells and thereby protects the host from lethal enteropathogenic infection [70,71] (Figure 2).

IBD also leads to progressive destruction of intestinal crypts, which induces a reduction of goblet cells and destruction of the intestinal mucosal layer. Interestingly, *Propionibacterium freudenreichii* KCTC 1063, a probiotic strain that produces propionate as a major metabolite, restores intestinal goblet cells by upregulating *muc2* expression at the transcriptional and translational level in vivo and ameliorates the DSS-induced acute colitis in a rat model [72].

Recent studies confirm the potential on the efficacy of fecal microbiota transplantation (FMT) to treat recurrent *Clostridium difficile* infection in patients with IBD. However, despite the fact that FMT improves some biological and endoscopic CD severity index scores, a low similarity index of patient and donor microbiota was found, suggesting that a single FMT may not be sufficient to induce significant changes in these patients [73].

Another strategy proposed to reverse dysbiosis in IBD patients is the use of specific dietary supplements. Thus, a diet low in fermentable oligosaccharides, disaccharides, monosaccharides, and polyols (FODMAP) diet (LFD), in a cohort of 67 patients, reduces fecal calprotectin and only significantly improved overall IBD-Q (disease-specific questionnaire on patient quality of life) scores in the short term [74].

On the other hand, 40 patients with UC that received, for 8 weeks, a symbiotic composed of six probiotic strains (*Enterococcus faecium, Lactobacillus plantarum, Streptococcus thermophilus, Bifidobacterium lactis, Lactobacillus acidophilus, Bifidobacterium longum*) and FOS (a prebiotic, NBL Probiotic Optima), showed an improvement in clinical and endoscopic activity and a reduction in serum C-reactive protein (CRP). However, no significant changes were found in other inflammatory markers (levels of hemoglobin, leukocytes, neutrophils, lymphocytes, and thrombocytes, as well as the neutrophil-lymphocyte ratio) [75].

### 6.2. Irritable Bowel Syndrome

**Figure 3:**
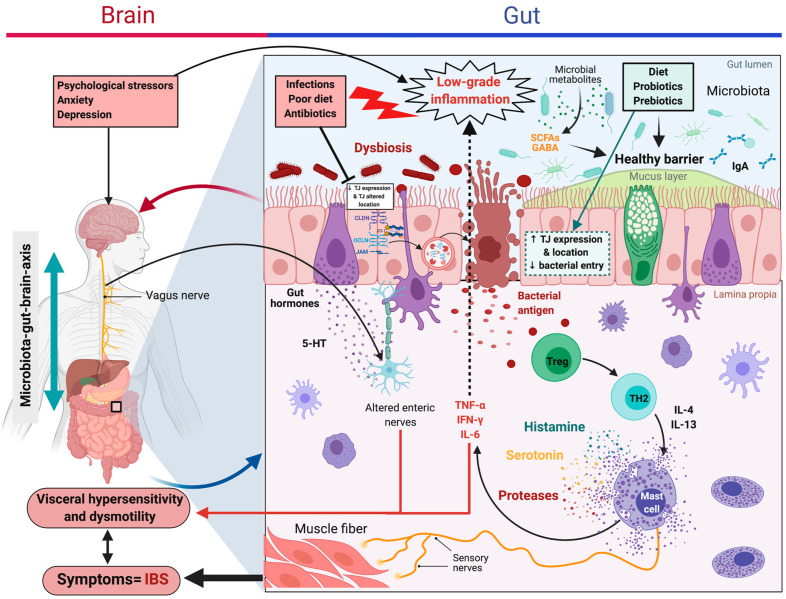
Increased intestinal permeability in irritable bowel syndrome (IBS). Main interactions described in IBS: different stimuli (e.g., bacterial or food antigens) can increase intestinal permeability through degradation or internalization of certain binding proteins that trigger MCs activation. These cells secrete pro-inflammatory cytokines that aggravate the altered permeability. In addition, histamine, serotonin, and proteases released by degranulated MCs are responsible for nerve excitation and the subsequent abdominal discomfort, pain, and intestinal transit changes. Microbiota of IBS patients shows lower biodiversity or an altered composition of microbes; thus, the use of probiotic strains, prebiotics, or synbiotic combinations has become a potential therapy to improve the composition of the intestinal microbiota. Their anti-inflammatory activities are also beneficial to IBS patients. (This figure was created with Biorender.com, access date: 24 May 2021).

IBS is one of the most common gastrointestinal disorders in the world [76]. It is characterized by recurrent discomfort or pain associated with changes in bowel movements. IBS diagnosis is established using a symptom-based approach, which distinguishes four subtypes (i) IBS-C, predominantly constipation; (ii) IBS-D, with diarrhea; (iii) IBS-M, mixed; and (iv) IBS-U, not subtyped [77]. More recently, a condition known as post-infection IBS (PI-IBS) was established as a new category of IBS, when acute infectious gastroenteritis represents the strongest known risk factor for IBS development [76]. The underlying mechanisms are still poorly understood, but several studies have demonstrated, using in vivo and ex vivo approaches, that altered intestinal permeability is associated with low-grade inflammation in all IBS subtypes. The primary mechanisms appear to be related to the regulation of tight and adherent junction proteins, OCLN in particular [78,79]. In a healthy context, OCLN is regulated by phosphorylation, but in IBS-D, it is dephosphorylated, which causes it to be internalized into the cytoplasm [78]. Furthermore, interactions with the ZO adherent junction proteins, especially ZO-1, promote the internalization of OCLN and a subsequent increase in paracellular permeability [78] (Figure 3). In addition, overexpression of the MLCK protein is observed in these patients, which induces increased phosphorylation of MLC and promotes the disruption of barrier integrity [80]. The increase in permeability facilitates the passage of luminal antigens that, in response, activate cells of the immune system, specifically mast cells (MCs) [81]. During their activation, MCs secrete granular compounds, such as pro-inflammatory cytokines (TNF-α, IFN-γ, IL-6, among others), or enzymes participating in pathophysiological processes [81]. The resulting low-grade inflammation could be responsible for visceral hypersensitivity, a feature of IBS associated with abdominal discomfort and/or pain [82]. Indeed, through mediators released from degranulated MCs, such as histamine, serotonin, or proteases, the nerves present in the intestine are directly excited, and the muscles relaxed, leading to abdominal pain and transit changes [83] (Figure 3). In this syndrome, many factors (altered microbiota, diet, stress, and age) have been implicated in the manifestation of symptoms, with each potentially driving a different underlying pathway affecting overall gut homeostasis [84].

There is accumulating evidence that the intestinal microbiota of IBS patients hosts a significantly lower biodiversity (or altered species composition) of microbes [85], with lower concentrations of bifidobacteria, lactobacilli, and *F. prausnitzii* compared with healthy controls [86]. Therefore, improvement of the composition of the intestinal microbiota has become a target of IBS treatment, with the use of probiotic strains, prebiotics, or synbiotic combinations. Most often, such treatments feature probiotic *Lactobacillus* species. For example, *Lactobacillus rhamnosus* GG was clinically shown to improve symptoms in IBS patients and prevent epithelial barrier dysfunction in human enteroid and colonoid cultures, specifically by restoring OCLN and ZO-1 expression to healthy control levels [87]. In a randomized controlled trial, IBS patients who received *L. acidophilus* DDS-1 and *Bifidobacterium lactis* UABla-12 exhibited normalization of stool type, in concert with improved outcomes in abdominal pain severity (numeric rating scale). These results were attributed to the previously demonstrated immune-regulatory and anti-inflammatory roles of the two probiotic strains [88]. Other protective effects have been described for *Clostridium butyricum*, a butyric acid-producing Gram-positive anaerobe found in the human intestine. Administration of *C. butyricum* was noted to improve overall IBS symptoms related to changes in the quality of life and bowel habit, but not abdominal pain and bloating. Treatment with *C. butyricum* may improve fatty acid metabolism (specifically, downregulating pathways linked to fatty acid metabolism, beta-alanine metabolism, tryptophan metabolism, and increasing levels of SCFAs in IBS patients) through suppressing the proliferation of *Clostridium sensu stricto*, a representative cluster of genus *Clostridium* that is frequently overabundant in fecal samples of IBS patients [89]. Another potential probiotic strain, *F. prausnitzii*, and its SN have demonstrated anti-inflammatory effects in chemically induced models of both acute and chronic colitis [55]. Similarly, *F. prausnitzii* was noted to reduce colonic sensitivity in a rodent model of IBS-like symptoms induced by neonatal maternal separation stress. The authors associated these results with the ability of *F. prausnitzii* to preserve TJ integrity [90].

Another strategy for the alleviation of IBS symptoms could be the use of prebiotics. Often, IBS patients are prescribed the FODMAP diet to improve their symptoms; this involves restricting the intake of certain sugars that may cause intestinal distress, such as fermentable oligosaccharides, disaccharides, monosaccharides, and polyols. However, this diet also reduces fecal concentrations of probiotic bifidobacterial, which have also been associated with the amelioration of IBS symptoms, likely due to a reduction in the availability of carbohydrates with prebiotic potential, such as β-GOS prebiotic [91]. In addition to or instead of dietary restrictions, recent research has focused on foods that can be added to patients’ diets to improve symptoms. For example, a randomized, double-blind study of a Norwegian cohort demonstrated the positive effect of lacto-fermented sauerkraut, which contains a variety of naturally occurring lactic acid bacteria. IBS patients who ate sauerkraut reported a reduction in total IBS symptom severity score (IBS-SSS) and demonstrated alterations in their gut microbiota. Members of order Clostridiales were less prevalent compared to baseline data, and beneficial sauerkraut-related strains (*L. plantarum* and *L. brevis*) were significantly more abundant in feces [92].

Similarly, a study of a novel prebiotic, trans-GOS, reported a significant enhancement in fecal concentrations of bifidobacteria and noted improvements in IBS sufferers’ symptoms, specifically with respect to stool consistency, flatulence, composite scores (abdominal pain/discomfort, bloating/distension, and bowel movement difficulty), and subjective global assessment values [93]. As prebiotics are fermented to varying degrees by different types of beneficial bacteria (e.g., bifidobacteria, lactobacilli), the immune-modulatory properties of prebiotic supplements have traditionally been thought to be microflora-dependent [94]. However, certain prebiotics have been found to bind directly to specific receptors of immune cells [95]. Likewise, non-digestible carbohydrates were reported to have a number of immune-modulatory effects, including increasing the production of T-helper 1 (Th1) and T-helper 2 (Th2) cytokines; increased expression of CD25, a marker for activated or regulatory T cells; enhancing IL-10 production in spleen cell cultures; enhancing spleen cell NK activity; and decreasing the ratio of CD4+/CD8+ T cells, among others [94].

Synbiotics have demonstrated potential as a promising approach for the treatment of functional bowel disorders, including IBS. A recent randomized, double-blind, placebo-controlled study of adult IBS patients indicated that a synbiotic preparation of probiotic strains of *Lactobacillus* (*L. rhamnosus* FloraActive™19070-2, *L. acidophilus* DSMZ 32418) and *Bifidobacterium* (*B. lactis* DSMZ 32269, *B. longum* DSMZ 32946, *B. bifidum* DSMZ 32403) together with short-chain FOS (scFOS) had beneficial effects in the treatment of IBS-D [96]. The primary endpoints considered were assessments of symptom severity with IBS-SSS, an improvement of IBS symptoms using the global improvement scale (IBS-GIS), and adequate relief of symptoms after four and eight weeks of therapy. An earlier study found that increasing doses of scFOS were associated with an increase in concentrations of fecal bifidobacteria [97], which have beneficial effects on colonic pH regulation, favorable immune modulation, pathogen exclusion, increasing SCFA production, and anti-protease production [98]. In general, the consumption of synbiotics by IBS patients has been associated with improvements in overall IBS/health-related quality of life (outcomes: frequency and severity of pain, bloating severity, bowel movement frequency, and satisfaction). In a clinical study, the combination of *L. acidophilus* La-5, *B*. *animalis* ssp. *lactis* BB-12, *Streptococcus thermophilus*, and dietary fiber (90% inulin, 10% oligofructose) had a short-term effect on the amount and proportion of La-5-like strains and *B. animalis* ssp. *lactis* in the fecal microbiome of IBS patients [99].

### 6.3. Food Allergy

FA is a growing and alarming disease that affects the quality of life of up to 10% of the population worldwide, with young children being the most affected group. Industrialized countries have a higher prevalence of FA than developing countries, although the incidence in the latter is increasing [100,101]. The loss of oral tolerance to food antigens leads to the inadequate production of allergen-specific IgE under a Th2 environment and the recruitment of MCs and other inflammatory cells, such as eosinophils, into the gastrointestinal mucosa [102]. Mediators released by MCs in response to allergen encounters are responsible for the early manifestations of FA, with diarrhea as the most common intestinal symptom [103].

**Figure 4:**
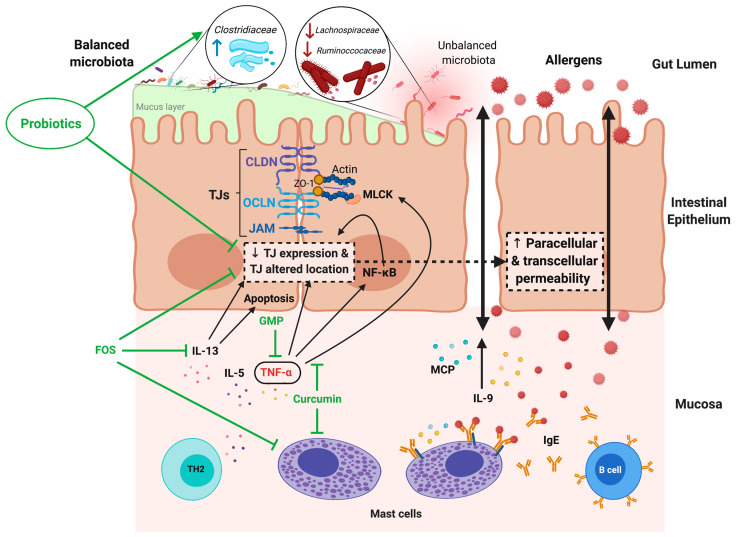
Increased intestinal permeability in food allergy (FA) and modulatory effects of probiotic, prebiotic, and food components. In the intestinal mucosa of allergic subjects, an inflammatory response develops that is dominated by Th2 cytokines, such as IL-5 and IL-13, which induce MC hyperplasia and allergen-specific IgE synthesis by B lymphocytes. In response to allergen recognition by IgE, which is bound to specific receptors on the MC surface, cells are activated and release TNF-α, proteases (MCP), and IL-9, as well as other inflammatory mediators. This allergic inflammatory environment induces a decrease in TJ expression and modifications that initiate an increase in the transcellular permeability of the mucosal epithelium. The augmented transcellular passage of allergens through enterocytes is also described during FA. Inflammatory mediators and mechanisms involved in the increased intestinal permeability during FA are indicated by black arrows. Treatment based on the oral intake of probiotic bacteria, prebiotics, and food components has shown beneficial effects in preventing or restoring the impaired barrier function in FA. The target molecules of these therapies are indicated by green lines. (This figure was created with Biorender.com, access date: 24 May 2021).

FA has been largely associated with alterations in intestinal mucosal permeability [104,105,106], which represents the main route of pathogenesis and causes some of the disease symptoms, such as diarrhea. This alteration can compromise the barrier function, affecting important intestinal tasks, such as nutrient hydrolysis or absorption [107]. In animal models of FA, oral sensitization to ovalbumin (OVA) induced an increase in the lactulose/mannitol (La/Ma) ratio (indicative of increased intestinal permeability) in excreted urine after two weeks of allergen intake, followed by an increase in serum anti-OVA IgE and a downregulation of ZO-1, claudin-2, claudin-8, and claudin-15 gene expression but upregulation of claudin-9 [106]. Similarly, rats with FA to OVA demonstrated downregulation of ZO-1, occludin, and claudins 1, 3, 5, 7, 8, 9, and 15 in intestinal tissue; which was accompanied by a widening and shortening of tight and adherent junctions, an increase in excreted La/Ma ratio, and a relocation of some TJ proteins in enterocytes [108]. The increase in intestinal permeability has been linked to intestinal mastocytosis and IL-9 overexpression (Figure 4), which are associated with a predisposition to clinical manifestations, such as diarrhea, in the absence of allergen sensitization [104]. MC proteases are also related to changes in paracellular permeability [109,110]. In allergic patients, studies have suggested that the increase in intestinal permeability may be the cause, and not just a consequence, of the effector phase of the allergic reaction [111,112]. Indeed, patients going on a specific elimination diet for at least six months showed increased intestinal permeability, which was strictly correlated with the severity of clinical symptoms [111]. In children with FA and on an allergen-free diet, intestinal hyperpermeability was also associated with limited growth [112]. Likewise, in children allergic to cow’s milk, large molecules, such as certain allergens, were able to pass through the intestine more readily during the effector phase of FA, as shown by a significantly higher transepithelial flux of horseradish peroxidase (HRP) in jejunal biopsies from allergic children compared to those from controls [113]. The HRP flux returned to control values after children were on a cow milk-free diet for several months.

Several cytokines that are abundant in the allergic inflammatory environment during FA are associated with changes in intestinal permeability. For example, TNF-α is an MC-derived cytokine that has been linked to allergic inflammation [114]. TNF-α increases the activity and the expression of MLCK [115,116], an enzyme that induces TJ regulation through MLC phosphorylation and a subsequent size-selective increase in barrier permeability (Figure 4) [117]. Additionally, IL-13, a type-2 cytokine highly involved in FA pathophysiology [103], was also implicated in changes in barrier permeability, specifically by increasing epithelial cell apoptosis and upregulating expression of the pore-forming TJ protein claudin-2 [118].

As discussed above, in the contexts of IBD and IBS, the intestinal microbiota makes major contributions to intestinal barrier function. In this sense, reduced bacterial diversity and lower counts of lactobacilli and bifidobacterial were detected in the gut of allergic children [119,120]. When *Lactobacillus salivarius*, *Lactibacillus paracasei*, *B. animalis*, and *B. bifidum* were orally administrated to infants, a significant reduction in the occurrence of atopic sensitization to common food allergens was shown [121]. Thus, there is a great deal of research aimed at evaluating the use of probiotics as potential treatments for FA, and preclinical studies using probiotics have investigated the mechanisms leading to the amelioration of FA outcomes. The beneficial effects of probiotics on immune balance, intestinal barrier function [122], and structure of intestinal microbiota results in an effective alleviation of allergic responses [123,124,125]. Thus, germ-free mice administered with a mixture of three probiotic strains (*L. rhamnosus* LOCK0900, *L. rhamnosus* LOCK0908, and *Lactobacillus casei* LOCK0919; isolated from stool samples from healthy infants) demonstrated stronger apical junctional complexes and adherent junctions in their enterocytes that resembled those of conventional mice and expressed higher levels of ZO-1 and occludin proteins than control germ-free mice [123]. Interestingly, after intestinal bacterial colonization by the three strains, mice showed a reduced allergic sensitization to the birch pollen allergen Bet v 1. Recently, it was shown that orally OVA-sensitized rats and administered with *C. butyricum* or *Lactobacillus reuteri* displayed decreased intestinal hyperpermeability, intestinal inflammation, eosinophil infiltration, and augmented villus length, in comparison with sensitized animals without treatment [102]. Furthermore, these probiotics prevented the downregulated expression and relocation of TJ proteins in enterocytes that was induced by allergen sensitization. An analysis of the microbiota composition in these animals found that the family *Clostridiaceae* was significantly enriched by treatment with either probiotic strain [102]. Likewise, a recent study about the effect of three lactobacillus strains (*L. plantarum* ZDY2013, *L. plantarum* WLPL04, and *L. rhamnosus* GG) on the intestinal barrier function of mice with FA showed an enhancement of TJ mRNA expression that was associated with the attenuation of allergic immune response and symptom severity [124]. In ZDY2013-treated animals, species in the families *Ruminococcaceae* and *Lachnospiraceae* were less abundant; two families that were previously reported significantly increased in the gut of infants allergic to cow’s milk [125].

Although the majority of meta-analysis and systematic reviews determine the positive impact of prebiotics to treat allergic conditions in humans [126,127], further rigorous trials considering FA in infants are required. Preclinical studies have shown that prebiotics administration is a potential treatment for FA. Using the transgenic mouse OVA23-3, which develops intestinal allergic inflammation in response to OVA in the diet, it was shown that the dietary intake of FOS decreased the Th2 cytokine response in intestinal immune tissues during the development of FA [128]. In addition, animals with an OVA allergy who were fed a diet supplemented with FOS demonstrated significant reductions in the number of MCs and the rate of edema formation in the duodenum compared with FA mice not given FOS [129]. The anti-allergenic functions of FOS might be linked to its protective effects on intestinal barrier function, which have been documented in multiple studies [130,131]. Similarly, experimental interventions with synbiotics seem promising for the development of prophylactic or therapeutic approaches to FA. Animals allergic to food components that received a diet enriched with *Bifidobacterium breve* M-16V and FOS or GOS reduced the magnitude of allergic effector response, showing a diminished MC degranulation and an increase in cecal levels of SCFAs, particularly butyric acid [132,133]. However, more research is needed in symbiotics application to FA, as results in clinical trials are still controversial. Allergic symptoms were reduced in children with FA receiving a symbiotic composed of oligofructose, long-chain inulin, acidic oligosaccharides, and *B. breve* M-16V [134], but no changes were observed in infants receiving *B. bifidum OLB6378* combined with FOS [135].

Finally, it is important to mention the favorable results of some food components in the prevention or treatment for FA. Glycomacropeptide (GMP) is a milk κ-casein-derived peptide with diverse biological activities ranging from prebiotic to metabolic, nutritional, antibacterial, and immunomodulatory effects [113]. Oral administration of GMP yielded beneficial effects on the health of animals with experimental inflammatory intestinal diseases [114,115,116,117], as well as in a pilot study of patients with distal UC [118]. GMP was able to downregulate the inflammatory pathway triggered by the binding of lipopolysaccharide (LPS) to the TLR4 receptor in cultured HT29-MTX cells [119]. GMP also upregulated biomarkers of epithelial integrity, such as the TJ proteins, in HT29-MTX or Caco-2 cells. Furthermore, GMP treatment increased transepithelial electrical resistance (TEER) in a TNF-α-challenged Caco-2/HT29-MTX co-culture monolayer [119], and significantly delayed the decrease in TEER that was induced by enterohemorrhagic and enteropathogenic *E. coli* strains in Caco-2 monolayers [120]. In the context of FA, prophylactic treatment with GMP decreased the allergic immune response and reduced the clinical manifestations, intestinal inflammation, and jejunum morphological alterations associated with experimental FA [121]. In particular, GMP treatment decreased the gene expression of TNF-α and upregulated that of TGF-β in the intestine of FA animals [121], and while TNF-α is a cytokine that increases intestinal permeability by downregulating ZO-1 expression through activating the NF-κB pathway [109], TGF-β is synthesized and released by cells in the intestine to preserve barrier function (Figure 4) [126,127]. On the other hand, curcumin is a yellow pigment in the Indian spice Turmeric (*Curcuma longa*) plant that can confer numerous health benefits [122]. In the cell lines Caco-2 and HT-29, curcumin was found to have an effect on LPS-activated signaling and TJ integrity. Specifically, it attenuated the inflammation-induced disruption of intestinal barrier function by reducing the IL-1β-mediated activation of p38 MAPK and, therefore, the expression of MLCK [123]. In a mouse model of OVA-induced intestinal anaphylaxis, curcumin prevented the development of mastocytosis, MC activation, and allergic manifestations, such as diarrhea; the last result was directly linked with the inhibition of NF-κB activation in the intestinal tissue of curcumin-treated allergic mice. In vitro assays confirmed that curcumin was able to inhibit the phosphorylation of the p65 subunit of NF-κB in bone marrow-derived MCs [124]. In addition, curcumin was recently reported to inhibit the catalytic activity of the protein disulfide isomerase, which is extracellularly expressed on the surface of MCs and participates in their activation and degranulation during FA, decreasing the production of TNF-α, IL-4, IL-6, and IL-13 by MCs in response to allergens [125].

## 7. Discussion

Modern life is intimately linked to changes in eating habits, which in turn impact the composition of the intestinal microbiota. Indeed, human evolution has been accompanied by incessant changes in lifestyle (including diet) [136,137,138]. Thus, humans evolved from being nomadic hunter-gatherers to a sedentary civilization practicing a new lifestyle based on agriculture and animal husbandry. This led to a drastic change in human eating habits (with the introduction of new foods, such as cereals and dairy products), which marked the beginning of the consumption of refined products, moving away from locally produced seasonal food. Thus, the modern diet in Westernized countries is characterized by a high consumption of animal products rich in sugars and fats, the use of preservatives, and a lower consumption of plant-based foods, such as fruits, vegetables, and whole grains [139]. Several studies have clearly demonstrated the impact of westernization on the gut microbiota of rural human populations, which have not yet adopted a western lifestyle. Indeed, these studies have shown that the gut microbiota of these rural populations (who consume high levels of fiber on a daily basis) is “enriched” in terms of microbial diversity compared to that of Western populations [140]. This depletion of microbial diversity in Western populations is accompanied by the loss of redundant microbial functions, which leads to jeopardizing the ability of the ecosystem to recover from exposure to external stressors (i.e., resilience), such as antibiotics and stress. The other consequence of reduced fiber intake on the microbiota is decreased production of SCFAs, such as acetate, propionate, and butyrate, which help inhibit the growth of many opportunistic pathogenic bacteria. In addition, Western dietary habits have been associated with the development of a plethora of chronic inflammatory diseases, some of which are linked to an altered gut barrier.

Different approaches are currently being investigated to correct abnormal host-microbe interactions, seal the broken barrier, and improve epithelial integrity, with the ultimate goal of complementing anti-inflammatory and immunologic therapies for patients with IBD, IBS, and PA. In addition, novel microbiome-focused interventions, such as probiotics, show promise. In line with the “common ground hypothesis”, a lot of effort has been devoted to designing therapies targeting dysbiotic bacterial communities and abnormal intestinal barriers as a treatment for intestinal barrier disorders. Further, diet-based approaches (e.g., prebiotics or other food components) that go beyond nutritional functions to modify microbe–microbe and microbe–host interactions could also be valuable tools to restore intestinal homeostasis and barrier integrity. Therefore, understanding the core interactions between gut microbiota and host barriers in the early subclinical stage will shed light on new therapeutic approaches for gut barrier pathologies.

Abnormal intestinal permeability appears to play a critical role in the development or maintenance of intestinal and extraintestinal diseases and has been linked with a variety of pathological conditions [4]. Multiple environmental factors affect intestinal integrity, including dietary lifestyle and drug and antibiotic administration, among others. It is now well accepted that alterations of the intestinal barrier are associated with chronic intestinal disorders such as IBS and IBD and with other pathologies, such as FA, obesity, and diabetes. Normal permeability of the intestinal barrier is preserved via diverse interactions among its different layers. However, several factors can disturb this permeability, allowing the passage of toxins or pathogens from the lumen to the immune layer and leading to a state of low-grade inflammation [4]. Different strategies have been explored for the treatment of these pathologies, including the manipulation of gut microbiota and the consumption of food supplements (e.g., probiotics, prebiotics, and synbiotics).

Although many advances have been made recently to decipher the action mechanisms and biological effects of prebiotics, probiotics, and symbiotics to be used as treatments to restore the altered intestinal barrier that underlies in IBD, IBS and FA, more research is needed as many results in humans are still controversial. In addition, there are important factors to take into account when prebiotic, probiotic, or both combined as synbiotics are formulated. In the case of probiotics, the safety, identity, purity, and potency of the live microorganism should be clearly and accurately described according to the best available methods that meet applicable regulatory standards for the product category. Relative to prebiotics, the structure and purity of the substrate also should be stated and characterized by appropriate chemical analyses. It is important to highlight that due to differences that exist across animal species, promising results obtained in preclinical studies showing prebiotic, probiotic, and synbiotic efficacy, safety, and appropriate dosing should be later demonstrated for humans. Benefits that confer probiotics in a host are highly strain- and dose-specific. In addition, the fact that a probiotic and a prebiotic have independently evidenced health-promoting effects is not enough for considering that together as a symbiotic product, it will show the same properties; its health effects in the target host must be examined.

Recent works have suggested the use of food containing a compound source of fiber together with microorganisms, such as *Bacillus* spores, that are able to induce the release of a prebiotic substrate from the compound under simulated gastrointestinal conditions [141]. Some of the prebiotic substrates released, such as galacto-rhamnogalacturonan, or the probiotic activity reported to some *Bacillus* strains, have been associated with an increase in barrier function and the prevention of cytokine-induced barrier dysfunction [142]. The benefit of this therapeutical approach is that the prebiotic promoting and probiotic effects of *Bacillus* spores are combined with their biological properties, as they produce or contain some components with anti-inflammatory, antioxidant, antimicrobial, or anti-viral activities [143]. Nevertheless, more research is needed to demonstrate their efficacy in human pathologies.
